# Obstructive Sleep Apnea in Head and Neck Cancer Survivors: A Systematic Review and Meta‐Analysis

**DOI:** 10.1002/oto2.70186

**Published:** 2026-01-30

**Authors:** Om Chitnis, J. Joseph Caraway, Ngun Cer Chin, Anne Guadalupi, Katherine Karahalios, Grace Baisden, Nora Watson, Michael Orestes

**Affiliations:** ^1^ Division of Otolaryngology Brook Army Medical Center San Antonio Texas USA; ^2^ Division of Otolaryngology–Head and Neck Surgery Walter Reed National Military Medical Center Bethesda Maryland USA; ^3^ Department of Research Programs Walter Reed National Military Medical Center Bethesda Maryland USA; ^4^ Department of Surgery Uniformed Services University of the Health Sciences Bethesda Maryland USA

**Keywords:** head and neck cancer, obstructive sleep apnea, polysomnography, apnea‐hypopnea index

## Abstract

**Objective:**

The purpose of this study is to perform the first systematic review and meta‐analysis examining the risks of obstructive sleep apnea in head and neck cancer survivors with the goal of determining if there is a statistically significant increase in the risk of obstructive sleep apnea after treatment for head and neck cancer. Additionally, we sought to determine if specific treatment modalities are associated with an increased risk of obstructive sleep apnea.

**Data Sources:**

A comprehensive literature search was performed using Embase, Ovid MEDLINE, Web of Science, and Ovid All EBM Reviews.

**Review Methods:**

Included studies performed polysomnography in head and neck cancer patients before and/or after treatment. Random effects meta‐analyses were used to estimate overall prevalences of obstructive sleep apnea and apnea‐hypopnea index change before versus after head and neck cancer treatment.

**Results:**

The literature search returned 575 articles for initial review, of which 16 articles met criteria for inclusion and meta‐analysis (419 participants). The mean obstructive sleep apnea prevalence in head and neck cancer survivors was 83.7%. Mean prevalence before treatment was 79.9% [70.0%, 88.3%] and after treatment was 88.7% [82.3%, 94.0%]. In random effects meta‐analysis, patients had a statistically significant increase in apnea‐hypopnea index of 4.28 [0.46, 8.09] after treatment.

**Conclusion:**

There is a high prevalence of obstructive sleep apnea in head and neck cancer survivors independent of treatment modality. Therefore, we propose that all head and neck cancer survivors undergo routine validated screening for obstructive sleep apnea.

Head and neck cancers (HNC) include malignancies of the oral cavity, nasal cavity, pharynx, larynx, paranasal sinuses, and salivary glands, with squamous cell carcinoma (SCC) being the most common type of HNC. Risk factors vary based on type and location of malignancy but include alcohol consumption, tobacco use, HPV infections, and radiation exposure. Treatment is determined by malignancy type, location, histopathology, staging, and p16 status, and can include surgical resection, radiation, and/or chemotherapy.^1^ While an in‐depth discussion of treatment modalities is outside the scope of this article, it should be noted that the treatment of HNC often results in significant morbidity, with one potential side effect being obstructive sleep apnea (OSA). Numerous studies have demonstrated a high incidence of OSA in HNC survivors; however, results vary widely between studies with the percentage of HNC survivors with OSA ranging from 12% to 96%.^2^ Multiple systematic reviews have been performed on this topic, all of which commented on the high prevalence of OSA in patients with a history of HNC. Still, there was no clear consensus on whether certain treatment modalities were associated with an increased risk of OSA.^2^, ^3^, ^4^, ^5^ Only one meta‐analysis on OSA in HNC survivors has ever been performed, and it specifically examined the risk of OSA in patients who received radiotherapy. This meta‐analysis was hampered by small sample sizes and significant heterogeneity between studies and ultimately showed no significant increase in OSA before versus after radiotherapy.^6^

The mechanism by which treatment of HNC could result in OSA is unclear. Previous studies have proposed that the development of OSA in this patient population could be secondary to radiation‐induced mucosal edema, mucositis, lymphedema, fibrosis, or post‐surgical anatomic alterations.^7^ Regardless, it is well‐recognized within the literature that OSA has numerous negative health consequences, including but not limited to hypertension, coronary artery disease, stroke, type 2 diabetes mellitus, poor wound healing, reduced quality of life, and prolonged/complicated postoperative recovery.^8^ Additionally, OSA has been associated with an increased risk for the development of certain malignancies potentially secondary to tissue damage from chronic hypoxemia.^9^

Currently, there is a lack of consensus regarding the relationship between OSA and HNC. Additionally, there is a lack of consensus regarding which types of HNC and treatment modalities result in the greatest risk for OSA. Furthermore, there are no clear guidelines regarding early intervention and detection of OSA in HNC patients. We hypothesize that the various structural changes induced by treatment of HNC may increase the risk of developing OSA in a treatment‐dependent manner. Given the lack of consensus, we seek to perform the first systematic review and meta‐analysis to critically examine the relationship between HNC treatment and the risk of OSA in HNC survivors.

## Methods

### Guidelines

**Figure 1:**
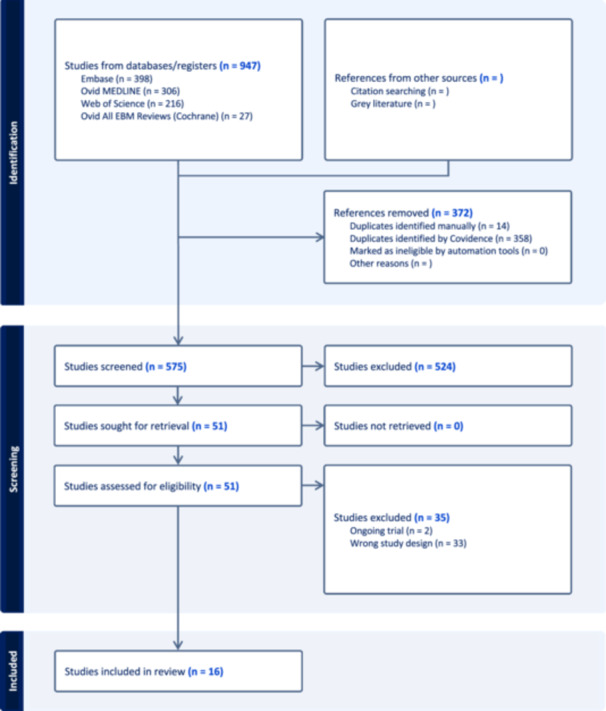
Preferred Reporting Items for Systematic Reviews and Meta‐Analyses (PRISMA) Diagram.

The authors adhered to the Preferred Reporting Items for Systematic Reviews and Meta‐analyses (PRISMA) strategy (see Figure 1).^10^ A Population, Intervention, Comparison, Outcome, Studies (PICOS) statement was utilized to identify studies that met inclusion or exclusion criteria.

### Search Strategy

An electronic literature search was performed using Embase, Ovid MEDLINE, Web of Science, and Ovid All EBM Reviews. The search was performed on 11/5/2023 with filters for peer‐reviewed and English‐language articles. The search strategy used the string “head and neck cancer” or “oral” or “lip” or “pharynx” or “larynx” or “oropharynx” or “tongue” or “salivary gland” or “tonsil” or “trachea” in combination with the string “sleep apnea” or “obstructive sleep apnea” or “OSA”. The authors did not identify any manuscripts via citation searching or in the gray literature. The exact search criteria used for each database are listed in Table 1.

**Table 1:** Database Search Terms Used for Systematic Review

Search terms	
Ovid MEDLINE	(exp “Head and Neck Neoplasms”/OR ((head OR neck OR esophageal OR oesophageal OR esophagus OR oesophagus OR face OR facial OR mouth OR oral OR gingival OR leukoplakia OR lip* OR palate OR palatal OR salivary‐gland* OR parotid OR sublingual OR submandibular OR tongue OR otorhinolaryngologic OR laryngeal OR larynx OR pharyngeal OR pharynx OR nose OR nasopharynx OR nasopharyngeal OR oropharynx OR oropharyngeal OR hypopharynx OR hypopharyngeal OR tonsil* OR tonsillar OR sinus OR paranasal OR maxillary OR trachea OR tracheal OR thyroid OR parathyroid) adj5 (cancer* OR neoplasm* OR carcinoma* OR tumor* OR tumour* OR squamous‐cell OR scc OR melanoma*)).ab,ti,kw.) AND (sleep apnea, obstructive/OR (apnea OR apnoea*).ab,ti,kw.) AND english.lg.
Embase	(‘head and neck tumor’/syn OR ((head OR neck OR esophageal OR oesophageal OR esophagus OR oesophagus OR face OR facial OR mouth OR oral OR gingival OR leukoplakia OR lip* OR palate OR palatal OR salivary‐gland* OR parotid OR sublingual OR submandibular OR tongue OR otorhinolaryngologic OR laryngeal OR larynx OR pharyngeal OR pharynx OR nose OR nasopharynx OR nasopharyngeal OR oropharynx OR oropharyngeal OR hypopharynx OR hypopharyngeal OR tonsil* OR tonsillar OR sinus OR paranasal OR maxillary OR trachea OR tracheal OR thyroid OR parathyroid) NEAR/5 (cancer* OR neoplasm* OR carcinoma* OR tumor* OR tumour* OR squamous‐cell OR scc OR melanoma*)):ab,ti,kw) AND (‘obstructive sleep apnea’/syn OR (apnea OR apnoea*):ab,ti,kw) AND ([article]/lim OR [article in press]/lim OR [review]/lim) AND [english]/lim
Web of Science	((head OR neck OR esophageal OR oesophageal OR esophagus OR oesophagus OR face OR facial OR mouth OR oral OR gingival OR leukoplakia OR lip* OR palate OR palatal OR salivary‐gland* OR parotid OR sublingual OR submandibular OR tongue OR otorhinolaryngologic OR laryngeal OR larynx OR pharyngeal OR pharynx OR nose OR nasopharynx OR nasopharyngeal OR oropharynx OR oropharyngeal OR hypopharynx OR hypopharyngeal OR tonsil* OR tonsillar OR sinus OR paranasal OR maxillary OR trachea OR tracheal OR thyroid OR parathyroid) NEAR/5 (cancer* OR neoplasm* OR carcinoma* OR tumor* OR tumour* OR squamous‐cell OR scc OR melanoma*)) AND (apnea OR apnoea*)
	Select Filters: Document Types: Article, Review Article and Early Access; Languages: English
Ovid All EBM Reviews	(exp “Head and Neck Neoplasms”/OR ((head OR neck OR esophageal OR oesophageal OR esophagus OR oesophagus OR face OR facial OR mouth OR oral OR gingival OR leukoplakia OR lip* OR palate OR palatal OR salivary‐gland* OR parotid OR sublingual OR submandibular OR tongue OR otorhinolaryngologic OR laryngeal OR larynx OR pharyngeal OR pharynx OR nose OR nasopharynx OR nasopharyngeal OR oropharynx OR oropharyngeal OR hypopharynx OR hypopharyngeal OR tonsil* OR tonsillar OR sinus OR paranasal OR maxillary OR trachea OR tracheal OR thyroid OR parathyroid) adj5 (cancer* OR neoplasm* OR carcinoma* OR tumor* OR tumour* OR squamous‐cell OR scc OR melanoma*)).ab,ti,kw.) AND (sleep apnea, obstructive/OR (apnea OR apnoea*).ab,ti,kw.) AND english.lg.

### Study Selection and Eligibility Criteria

Studies were included based on the PICO criteria listed in Table 2. Exclusion criteria were non‐English studies, studies that did not evaluate all included patients with a formal sleep study, studies that included patients with a prior history of OSA, studies that prescreened patients to select subjects, or studies that used a nonstandard definition for OSA (ie, AHI ≥ 5). Titles and abstracts were screened by 2 independent reviewers (OC, NC) with the help of a third reviewer (JC) to resolve conflicts. The full text of all identified studies were retrieved and underwent further screening to confirm that they met inclusion criteria. A PRISMA flow diagram outlining the steps of study selection is presented in Figure 1. The literature search and study selection were performed using the Covidence systematic review management software (Melbourne, Australia).

**Table 2:** PICOS Statement and Inclusion Criteria

Population	Patients with head and neck cancer with no prior history of OSA
Intervention	HNC treatment including chemotherapy, radiation, and/or surgery (all types)
Comparison	Sleep study results (PSG and/or HSAT)
Outcomes	AHI scores after treatment
Studies	Prospective and retrospective cohort studies, cross‐sectional studies, randomized control trials

Abbreviations: AHI, apnea‐hypopnea index; HNC, head and neck cancer; HSAT, home sleep apnea test; PSG, polysomnography.

### Data Collection

Three reviewers (OC, NC, KK) manually extracted data and a fourth reviewer (JC) cross‐checked extracted data for accuracy. Data extracted from each study included study design, sample size, patient demographics, cancer type/location, treatment modalities, and pre/posttreatment AHI scores.

### Quality Assessment

The authors assessed the level of evidence for each article using the Oxford Centre for Evidence‐Based Medicine March 2009 guidelines which utilizes a numeric scale of 1 to 5.^11^ Bias was assessed using the validated Methodological Index for Non‐Randomized Studies (MINORS) criteria that includes 12 items each scored as 0 for not reported, 1 for reported but inadequate, and 2 for adequate.^12^ Articles were reviewed by 2 authors (OC, JC) and scored out of 24, with higher scores indicating a lower risk of bias. The final score was an average of the total scores of 2 independent reviewers (see Table 3).

**Table 3:** Summary of general characteristics, demographics, quality criteria, and results of all included studies

Author (year)	N	MINORS criteria^a^	Demographics	Study design	Results
Chan et al. 2012	26	21	HNC Type: Tongue Location: Taiwan Study Setting: single‐center Study Type: retrospective cross‐sectional study Mean Age (HNC group): 52 Sex (HNC group): 8% female, 92% male Mean BMI: 25.2 kg/m^2^	Treatment Modalities: − Surgery alone, 9 (35%) − Surgery + RT, 14 (65%) Patients underwent PSG 6 months to 11 years after treatment.	− 14 of 26 patients (53.8%) had OSA after treatment. − Mean AHI after treatment: − Overall: 8.96 − Surgery alone: 9.64 − Surgery + RT: 8.6
Chen et al. 2023	59	20	HNC Type: Larynx Location: China Study Setting: single‐center Study Type: prospective cohort Mean Age (HNC group): 60.1 Sex (HNC group): 4% female, 96% male Mean BMI: 22.9 kg/m^2^	Treatment Modalities: − Surgery alone, 45 (76.3%) − Surgery + RT, 12 (20.3%) − Surgery + CRT, 2 (3.4%) Patients underwent HSAT 1 to 17.5 months (average 4.3 months) after treatment.	− 44 of 59 patients (74.6%) had OSA after treatment. − Mean AHI after treatment: − Overall: 12.8
Gil et al. 2024	47	21	HNC Type: Oropharynx, hypopharynx, larynx Control: Study of Health in Pomerania Cohort Location: Germany Study Setting: single‐center Study Type: prospective cohort Median Age (control group): 54 Sex (control group): 46% female, 54% male Mean Age (HNC group): 63.4 Sex (HNC group): 8.5% female, 91.5% male Mean BMI: 26.2 kg/m^2^ before treatment, 23.6 kg/m^2^ after treatment	Treatment Modalities: − CRT, 12 (52.2%) − Surgery + adjuvant therapy, 11 (47.8%) Patients underwent PSG and ESS before treatment, and PSG 2‐11 months (average 5 months) after treatment.	− 33 of 47 patients (70%) had OSA before treatment. − 20 of 23 patients (87%) who followed up had OSA after treatment. − Mean AHI before/after treatment: − Overall: pretreatment 11.6; posttreatment 19.1. − CRT Group: pretreatment 11.8; posttreatment 22.31. − Surgery + adjuvant therapy: pretreatment 11.4; posttreatment 15.6.
Gilat et al. 2013	15	21	HNC Type: Tongue Location: Israel Study Setting: single‐center Study Type: prospective cohort Mean Age (HNC group): 57 Sex (HNC group): 67% female, 33% male Mean BMI: 24.1 kg/m^2^	Treatment Modalities: − Surgery alone, 2 (13.3%) − Surgery + RT, 11 (73.3%) − Surgery + CRT, 2 (13.3%) Patients underwent PSG and ESS 2‐6 years (average 4.9 years) after treatment.	− 8 of 15 patients (53.3%) had OSA after treatment. − Mean AHI after treatment: − Overall: 8 − Surgery alone: 4.85 − Surgery + RT: 8.64 − Surgery + CRT: 8.5
Huang et al. 2021	15	22	HNC Type: Oral cavity, oropharynx Location: Taiwan Study Setting: single‐center Study Type: prospective cohort Mean Age (HNC group): 56.2 Sex (HNC group): 6.7% female, 93.3% male	Treatment Modality: − Surgery alone, 15 (100%) Patients underwent PSG before and 0.5 to 19 months (average 5.1 months) after treatment.	− 14 of 15 patients (93%) had OSA before treatment. − 15 of 15 patients (100%) had OSA after treatment − Mean AHI before/after treatment: − Surgery alone: pretreatment 40.7; posttreatment 37.3.
Huppertz et al. 2021	33	19	HNC Type: Oropharynx, hypopharynx, larynx Location: Germany Study Setting: single‐center Study Type: prospective cohort Mean Age (HNC group): 64.4 Sex (HNC group): 18% female, 82% male Mean BMI: 24.83 kg/m^2^ before treatment, 22.82 kg/m^2^ after treatment	Treatment Modalities: − Surgery + RT, 15 (45.4) − Surgery + CRT, 6 (18.2) − CRT alone, 12 (36.4) Patients underwent HSAT, ESS, and PSQI after treatment.	− 30 of 33 patients (90%) had OSA before treatment. − 16 of 17 patients (94.1%) who followed up had OSA after treatment. − Mean AHI before/after treatment: − Overall: pretreatment 20.2, posttreatment 20.4.
Huyett et al. 2017	16	21	HNC Type: Larynx, oropharynx Location: US Study Setting: single center Study Type: prospective cohort Median Age (HNC group): 61.6 Sex (HNC group): 18.8% female, 81.2% male Median BMI: 29.8 kg/m^2^	Treatment Modalities: − CRT, 14 (87.5%) − RT, 2 (12.5%) Patients underwent HSAT, ESS, FOSQ‐10, and UW‐QOL 0.86 to 8.3 years (average 2.9 years) after treatment.	− 8 of 16 patients (50%) had OSA after treatment. − Mean AHI after treatment: − Overall: 10.34
Inoshita et al. 2022	21	22	HNC Type: Nasopharynx, oropharynx, hypopharynx, larynx Location: Japan Study Setting: multi‐center Study Type: prospective cohort Mean Age (HNC group): 64.9 Sex (HNC group): 3.1% female, 96.9% male Mean BMI: 22.7 kg/m^2^ before treatment, 20.2 kg/m^2^ after treatment	Treatment modalities: − RT alone, 2 (9.5%) − CRT, 17 (81%) − BRT, 1 (4.8%) − IA‐CRT, 1 (4.8%) Patients underwent PSG or portable sleep testing before and after treatment.	− 17 of 21 patients (81%) had OSA before treatment. − 18 of 21 patients (85.7%) had OSA after treatment. − Mean AHI before/after treatment: − Overall: pretreatment 14.5, posttreatment 14.9
Israel et al. 2006	22	21	HNC Type: Larynx Location: Brazil Study Setting: single‐center Study Type: prospective cohort Mean Age (HNC group): 65.5 Sex (HNC group): 9.1% female, 90.9% male Mean BMI: 23.6 kg/m^2^	Treatment modality: Surgery alone, 22 (100%) Patients underwent PSG and ESS 4 months to 12.2 years (average 3.2 years) after treatment.	− 19 of 22 patients (86.4%) had OSA after treatment. − Mean AHI after treatment: − Surgery alone: 19.4
Liao et al. 2022	23	23	HNC Type: Oral cavity, oropharynx Location: Taiwan Study Setting: single‐center Study Type: prospective cohort Mean Age (HNC group): 54.7 Sex (HNC group): 100% male Mean BMI: 24.4 kg/m^2^ before treatment, 22.9 kg/m^2^ after treatment	Treatment modalities: − Surgery alone, 10 (43.5%) − Surgery + RT, 4 (17.4%) − Surgery + CRT, 9 (39.1%) Patients underwent PSG before and 6 months after treatment.	− 21 of 23 patients (91.3%) had OSA before treatment. − 22 of 23 patients (95.7%) had OSA after treatment. − Mean AHI after treatment: − Overall: pretreatment 23.3, posttreatment 34.6
Lin et al. 2014	18	24	HNC Type: Nasopharynx Location: Taiwan Study Setting: single‐center Study Type: prospective cohort Mean Age (HNC group): 49.8 Sex (HNC group): 16.7% female, 83.3% male Mean BMI: 24.7 kg/m^2^ before treatment, 21.8 kg/m^2^ after treatment	Treatment Modalities: − RT − CRT Patients underwent PSG, ESS, and VAS 6 months after treatment.	− 13 of 18 patients (72.2%) had OSA before treatment. − 14 of 18 patients (77.8%) had OSA after treatment. − Mean AHI before/after treatment: − Overall: pretreatment 26.2, posttreatment 21.7
Nguyen et al. 2021	20	21	HNC Type: Larynx Location: France Study Setting: single‐center Study Type: prospective cohort Mean Age (HNC group): 66 Sex (HNC group): 25% female, 75% male Mean BMI: 26.28 kg/m^2^	Treatment Modalities: − Surgery alone, 10 (50%) − Surgery + RT, 10 (50%) Patients underwent HSAT, ESS, and PSQI after treatment.	− 19 of 20 patients (95%) had OSA after treatment. − Mean AHI after treatment: − Overall: 24.6 − Surgery alone: 27.9 − Surgery+RT: 21.2
Ouyang et al. 2019	40	23	HNC Type: Larynx Location: China Study Setting: single‐center Study Type: prospective cohort Mean Age (HNC group): 55.5 Sex (HNC group): 7.5% female, 92.5% male Mean BMI: 23.33 kg/m^2^ before treatment, 23.56 kg/m^2^ after treatment	Treatment Modalities: − Surgery alone, 40 (100%) Patients underwent PSG and ESS before and 3 months after treatment.	− 23 of 40 patients (57.5%) had OSA before treatment. − 33 of 40 patients (82.5%) had OSA after treatment. − Mean AHI after treatment: − Surgery alone: pretreatment 6.57, posttreatment 14.34
Qian et al. 2010	24	21	HNC Type: Oral cavity, oropharynx Location: Canada Study Setting: Study Type: prospective cohort Mean Age (HNC group): 60. Sex (HNC group): 33.3% female, 66.7% male Mean BMI: 27.6 kg/m^2^ before treatment, 29.67 kg/m^2^ after treatment	Treatment Modalities: − Surgery alone, 3 (12.5%) − RT, 3 (12.5%) − CRT, 6 (25%) − Surgery + CRT, 12 (50%) Patients underwent PSG and ESS 6 to 69 months (average 25.6 months) after treatment.	− 23 of 24 patients (95.8%) had OSA after treatment. − Mean RDI after treatment: − Overall: 23.4 − Surgery alone: 16.7 − RT: 24.7 − CRT: 11.5 − Surgery + CRT: 30.8
Teixeira et al. 2013	14	21	HNC Type: Larynx Location: Brazil Study Setting: Single‐center Study Type: Cross‐sectional clinical study Mean Age (HNC group): 64.9 Sex (HNC group): 7% female, 93% male Mean BMI: 25.7 kg/m^2^	Treatment Modalities: − Surgery alone, 14 (100%) Patients underwent PSG, spirometry, and ESS 9 to 102 months (average 67.2 months) after treatment.	− 13 of 14 patients (92.9%) had OSA after treatment. − Mean AHI after treatment: − Surgery alone: 24
Yoshikawa et al. 2023	15	23	HNC Type: Oral cavity, oropharynx Location: Japan Study Setting: single center Study Type: Prospective case control Median Age (HNC group): 62 Sex (HNC group): 33% female, 67% male Median BMI: 21.3 kg/m^2^ before treatment, 19 kg/m^2^ after treatment	Treatment Modality: − Surgery alone, 15 (100%) Patients underwent portable type 4 sleep study before and 41 days (median) after treatment.	− 12 of 15 patients (80%) had OSA before treatment. − 12 of 15 patients (80%) had OSA after treatment. − Mean AHI before/after treatment: − Surgery alone: pretreatment 13, posttreatment 18.4

Abbreviations: AHI, apnea‐hypopnea index; BRT, bioradiotherapy; CRT, chemoradiotherapy; ESS, Epworth Sleepiness Scale; FOSQ‐10, Functional Outcomes of Sleep Questionnaire‐10; HNC, head and neck cancer; HSAT, home sleep apnea test; IA‐CRT, supra‐selective intraarterial concurrent chemoradiotherapy; PSG, polysomnography; PSQI, Pittsburgh Sleep Quality Index; RT, radiotherapy; UW‐QoL University of Washington Quality of Life Questionnaire; VAS, snoring visual analog scale.

^a^ Methodological Index of Nonrandomized Studies (MINORS) criteria^12^ : (1) a clearly stated aim; (2) inclusion of consecutive patients; (3) prospective collection of data; (4) endpoints appropriate to the aim of the study; (5) unbiased assessment of the study endpoint; (6) follow‐up period appropriate to the aim of study; (7) loss to follow‐up less than 5%; (8) prospective calculation of the study size; (9) an adequate control group; (10) contemporary groups, that is, groups studied within the same time period; (11) baseline equivalence of groups; and (12) adequate statistical analysis. Each component is rated out of 2, and the ideal global score is 24 for comparative studies.

### Meta‐Analysis

Random effects (RE) meta‐analysis was used to estimate the overall prevalence of OSA across all 16 studies that reported the prevalence of OSA in HNC patients after HNC treatment. Binomial (Clopper‐Pearson) confidence intervals were reported for individual studies, and individual proportions were transformed for meta‐analysis using the double arcsine transformation (Freeman‐Tukey) to improve the estimation of their sampling variances. Studies were weighted with the inverse variance method, and the DerSimonian‐Laird estimator was used to estimate the between‐study variance. These analyses were repeated to estimate OSA prevalences pre‐ and post‐HNC treatment among the eight studies that reported OSA prevalence both pre‐ and post‐HNC treatment.

RE meta‐analysis was also used to estimate the overall mean change in AHI across the eight studies that reported AHI in HNC patients before and after treatment. Mean AHI changes with their 95% confidence intervals were defined for individual studies as the post‐pre HNC treatment mean AHI score. Sampling variances calculated using an assumed 0.7 correlation coefficient for paired AHI measures, based on the average of the correlation coefficients calculated from individual level data reported by Ouyang et al. and Lin et al.^13^, ^14^ Additional RE meta‐analyses were done to estimate the overall posttreatment mean AHI across all treatment modalities, as well as subgroup analyses of each treatment modality (surgery alone; surgery + adjuvant therapy; or radiation ± chemotherapy) reported by the individual studies in which data reporting allowed for subgroup data extraction. Additionally, a meta‐regression was performed of AHI change with continuous pre‐age and pre‐BMI as separate moderators.

Heterogeneity across studies was reported as the *I* ^2^ statistic with statistical significance Analyses were performed using the meta^15^ and metafor^16^ packages in R.^17^

## Results

### Study Characteristics

The literature search returned 575 studies for title/abstract screening, of which 51 met criteria for full‐text review. 16 articles ultimately met inclusion criteria (Figure 1).^13^, ^14^, ^18^, ^19^, ^20^, ^21^, ^22^, ^23^, ^24^, ^25^, ^26^, ^27^, ^28^, ^29^, ^30^, ^31^ These included prospective cohort studies^13^, ^14^, ^19^, ^20^, ^21^, ^22^, ^24^, ^25^, ^26^, ^27^, ^28^, ^29^, ^30^, ^31^ and cross‐sectional studies^18^, ^23^ published from 2006 to 2024. All 16 articles reported posttreatment AHI scores (N = 419), and eight studies included both pretreatment and posttreatment sleep study results (N = 226).^13^, ^14^, ^24^, ^25^, ^27^, ^28^, ^30^, ^31^ The timeline at which sleep studies were conducted varied significantly both within and between studies, ranging from 2 weeks to 12.2 years posttreatment, with the average sleep study conducted at 14.8 months (see Table 3). The eight studies that included both pre‐ and posttreatment sleep study results conducted sleep studies 1 to 19 months post‐treatment with an average of 4.7 months posttreatment. It should also be noted that some studies reported AHI scores for individual patients,^13^, ^14^, ^20^, ^21^, ^22^, ^23^, ^26^, ^29^ while others reported a mean AHI score across all patients regardless of treatment modality.^18^, ^19^, ^24^, ^25^, ^27^, ^28^, ^30^, ^31^

Substantial heterogeneity (>50%, exceeding the within‐study sampling variability) was found for OSA prevalence (*I* ^2^ = 68%) and for overall mean AHI change (*I* ^2^ = 72.9%), challenging the interpretation of overall estimates. Bias was assessed via the MINORS criteria, with an average score of 21.5 and scores ranging from 19 to 24. Included studies are summarized in Table 3.

### Demographic Data

All 16 articles contained baseline demographic data. The mean age of patients was 51.1 and ranged from 27 to 90 years. Mean BMI was 24.2 kg/m^2^ and ranged from 14.82 to 47 kg/m^2^. In all studies, patients were predominantly male. Two articles were from studies performed in Brazil,^18^, ^21^ 1 from Canada,^22^ 2 from China,^13^, ^19^ 1 from France,^26^ 2 from Germany,^27^, ^28^ 1 from Israel,^29^ 2 from Japan,^30^, ^31^ 4 from Taiwan,^14^, ^23^, ^24^, ^25^ and 1 from the United States.^20^ Table 3 summarizes demographic information.

### OSA in HNC Survivors

**Figure 2:**
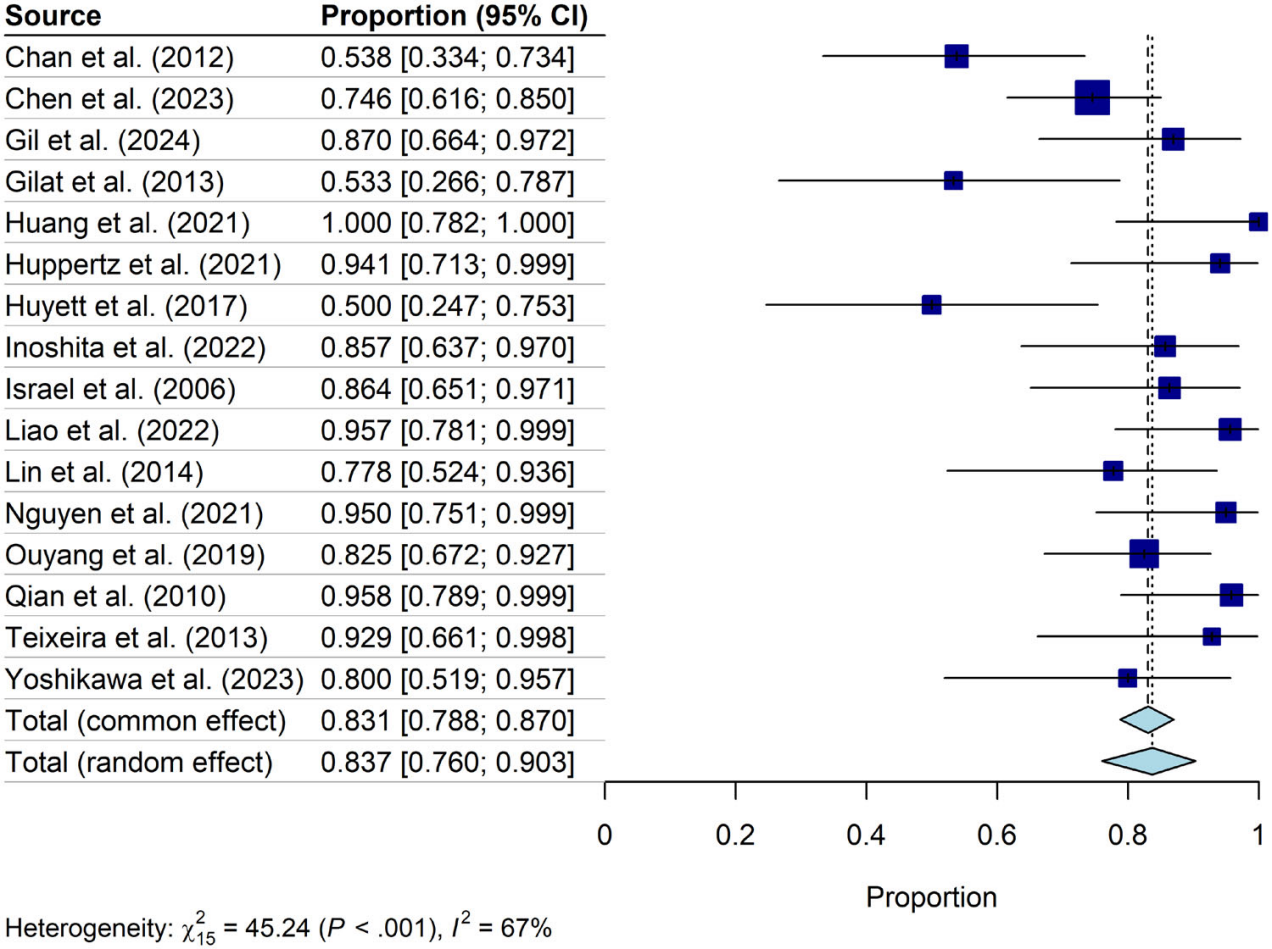
Prevalence of OSA after treatment within study groups. Square size indicates relative sample size. OSA, obstructive sleep apnea.

**Figure 3:**
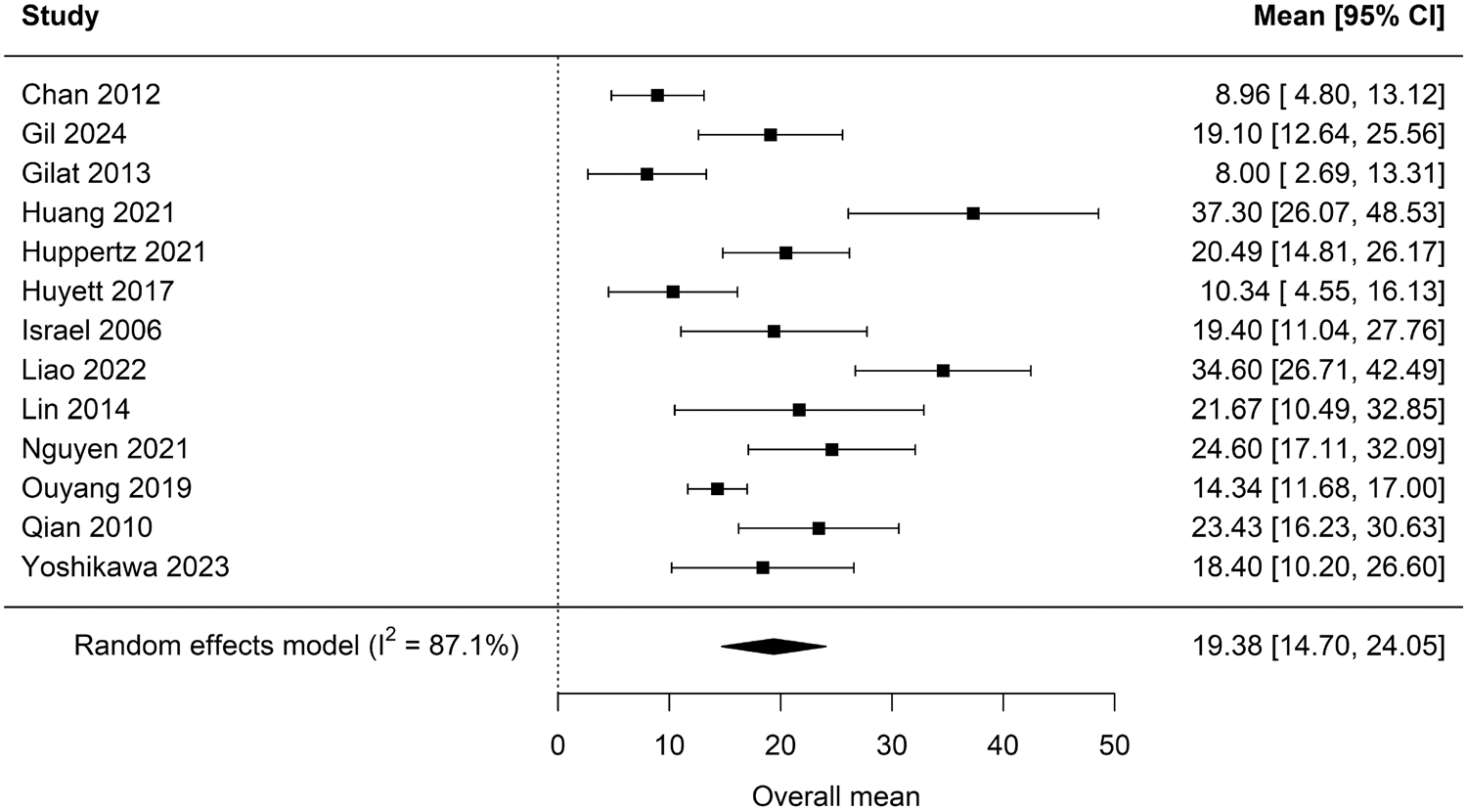
Mean AHI scores after treatment of HNC across all treatment modalities.

Data collected from all 16 studies were used to calculate the overall prevalence of OSA (AHI ≥ 5) in HNC survivors across all treatment modalities. Meta‐analysis revealed that the total RE prevalence of OSA across all HNC survivors was 83.7% [76.0%, 90.3%] (Figure 2). The mean RE AHI of HNC survivors across all studies and treatment modalities was 19.38 [14.70, 24.05] (Figure 3).

### Sub‐Group Analysis of AHI by Treatment Modality

**Figure 4:**
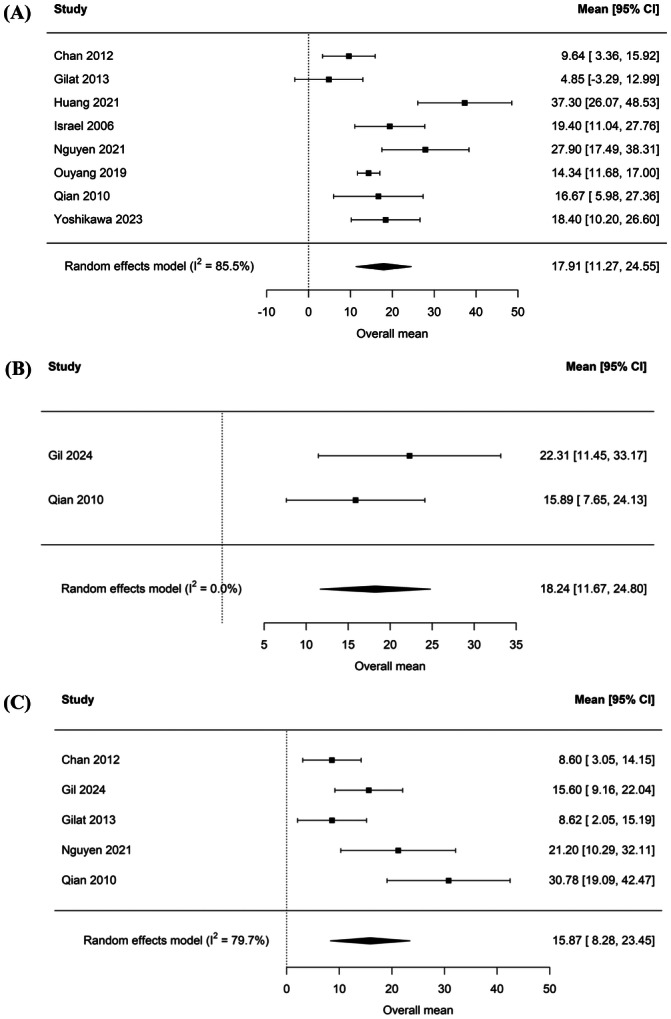
Subgroup analysis of mean AHI scores after (A) Surgery Alone (8 studies), (B) Chemoradiation (2 studies), or (C) Surgery with Adjuvant Therapy (5 studies).

An exploratory meta‐analysis was performed examining post‐treatment AHI in subgroups by treatment modality. Analysis was limited to studies that published individualized patient data based on treatment modality. Meta‐analysis of these studies revealed an average AHI of 17.91 [11.27, 24.55] in patients who underwent surgery alone (Figure 4A),^13^, ^21^, ^22^, ^23^, ^24^, ^26^, ^29^, ^31^ average AHI of 18.24 [11.67, 24.80] in patients who underwent radiation ± chemotherapy (Figure 4B),^22^, ^27^ and average AHI of 15.87 [8.28, 23.45] in patients who underwent surgery and adjuvant therapy (Figure 4C).^22^, ^23^, ^26^, ^27^, ^29^

### AHI Change After Treatment

**Figure 5:**
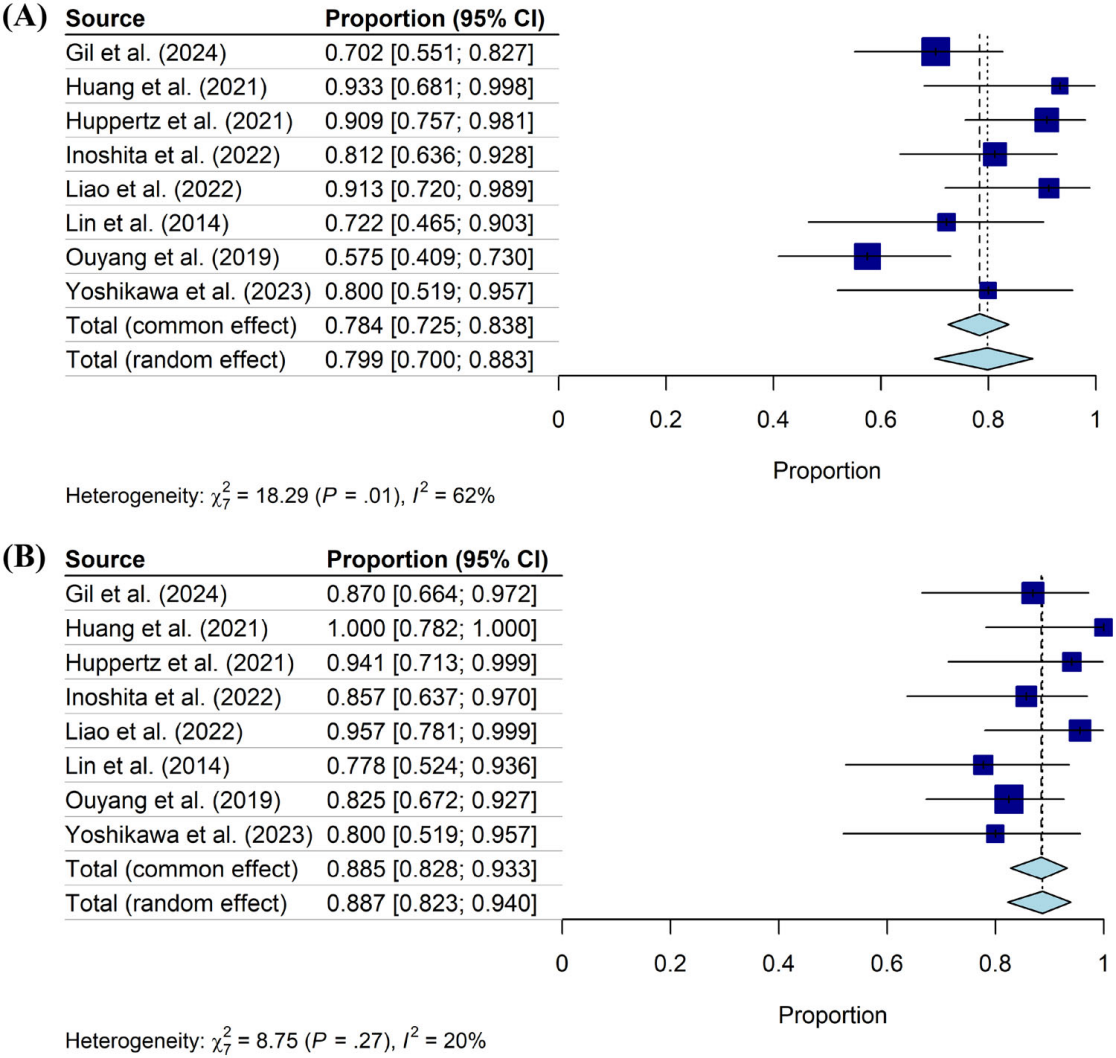
Prevalence of OSA (A) before and (B) after treatment (8 studies). Square size indicates relative sample size.

**Figure 6:**
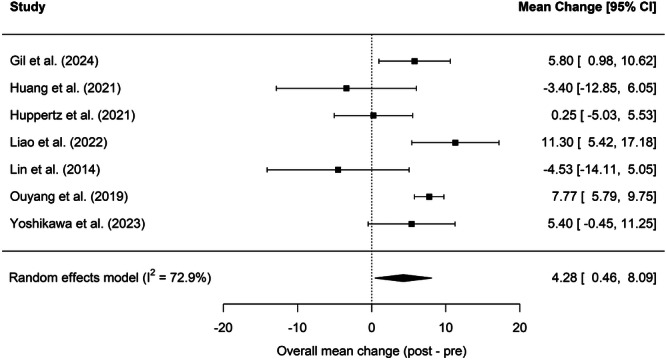
Overall mean change of AHI before versus after treatment of HNC. AHI, apnea‐hypopnea index; HNC, Head and neck cancers.

The 8 articles that reported sleep studies before and after treatment for HNC were included in a meta‐analysis assessing mean change in AHI pretreatment versus posttreatment.^13^, ^14^, ^24^, ^25^, ^27^, ^28^, ^30^, ^31^ The prevalence of OSA before treatment was 79.9% [70.0%, 88.3%] (Figure 5A) and the prevalence after treatment was 88.7% [82.3%, 94.0%] (Figure 5B). In RE meta‐analysis, HNC patients had a statistically significant increase in AHI of 4.28 [0.46, 8.09] after treatment (Figure 6). Notably, of the 16 included studies, 13 studies found no association between AHI and BMI,^13^, ^14^, ^19^, ^20^, ^21^, ^22^, ^25^, ^26^, ^27^, ^28^, ^29^, ^30^, ^31^ while 2 studies found a statistically significant correlation between AHI and BMI.^18^, ^23^ Additionally, a meta‐regression was performed examining the relationship between AHI and BMI, and ultimately no association was found between these variables.

### Screening Instruments Used to Detect OSA in HNC Survivors

10 articles included in this study routinely screened patients for OSA in conjunction with performing polysomnography.^13^, ^14^, ^18^, ^20^, ^21^, ^22^, ^26^, ^27^, ^28^, ^29^ All 10 articles used the Epworth Sleepiness Scale and other well‐validated screeners such as the STOP‐BANG Questionnaire (SBQ) were not employed. Three of these articles conducted a statistical analysis that evaluated ESS scores in relation to AHI. One study found a statistically significant correlation between AHI and ESS scores,^18^ while the two other studies found no statistically significant corelation.^22^, ^29^

## Discussion

Numerous prospective and cross‐sectional studies have demonstrated an association between HNC therapy (ie, Surgery, RT, and/or Chemotherapy) and OSA.^3^, ^4^, ^5^, ^6^ However, most of these studies only evaluated patients for OSA after HNC therapy. The 8 studies that assessed patients for OSA before and after HNC cancer therapy are observational and use small sample sizes, which may limit the generalizability of their results. The variability in tumor location, staging, treatments, and follow‐up times makes comparisons difficult. Previous reviews have found an association between HNC therapy and OSA but could not conduct a meta‐analysis due to study heterogeneity. Recent systematic reviews by Gavidia et al and Ralli et al reported a prevalence of OSA in HNC survivors ranging from 12% to 95.8% across included studies.^2^, ^3^ However, both systematic reviews included studies that used a variety of AHI and RDI cut‐offs as the diagnostic criteria for OSA rather than a standard cutoff. A previous systematic review conducted a meta‐analysis on OSA in HNC survivors, focusing only on those who received radiotherapy. However, this study was limited by small sample sizes and substantial heterogeneity.

Given the heterogeneity among studies there is no consensus regarding the following: (1) are HNC survivors at increased risk for developing OSA, (2) do specific HNC treatment modalities or combinations thereof result in increased risk of developing OSA, (3) how does the location or stage of HNC impact risk for development of OSA, (4) when should HNC survivors be screened for OSA. Additionally, the underlying mechanism for development of OSA following treatment of HNC remains unclear. It has been proposed that RT could lead to OSA via mucosal edema/mucositis in the initial weeks, followed by lymphedema over months, and ultimately fibrosis over the following months to years. Surgical treatment could result in anatomic alterations, scarring, poor muscle tone, and possible decrease of muscle function leading to OSA.^7^, ^18^, ^19^

We identified 16 studies that had evaluated for OSA in HNC survivors using AHI ≥5 as the diagnostic criteria for OSA. RE Meta‐analysis revealed an OSA prevalence of 83.7% in HNC survivors across all treatment types (see Figure 2). Nine of the 16 included studies also examined the prevalence of OSA based on treatment modality. Our meta‐analysis revealed a posttreatment average AHI of 17.91 [11.27, 24.55] in patients who underwent surgery alone, average AHI of 18.24 [11.67, 24.80] in patients who underwent radiation with or without chemotherapy, and average AHI of 15.87 [8.28, 23.45] in patients who underwent surgery and adjuvant therapy (see Figure 4). These posttreatment mean AHI scores indicate that surgery and radiotherapy independently may increase the risk of OSA in HNC survivors, but further research is needed to determine risk by treatment modality. 8 of the 16 included studies reported prevalence of OSA before and after HNC therapy. Meta‐analysis of these studies revealed a mean prevalence of OSA of 79.9% [70%, 88.3%] before therapy and 88.7% [82.3%, 94.0%] after therapy (see Figure 5). While the prevalences of OSA before and after HNC therapy were both high, overall prevalences did not quantitatively differ and confidence intervals largely overlapped. However, a meta‐analysis did reveal a statistically significant increase in AHI of 4.26 [0.46, 8.09] after HNC therapy (Figure 6). The statistically significant increase in mean AHI after therapy suggests that HNC therapy may increase the risk of OSA in HNC survivors.

Although this study was not specifically designed or sufficiently powered to statistically assess an increase in the prevalence of OSA among HNC survivors, future research with a larger sample size could investigate this potential objective. However, it is important to note that in the studies that measured pretreatment AHI, most patients already had OSA before undergoing HNC treatment. While most HNC patients, as well as most patients in the included studies, are at increased risk of OSA given age over 50 and male sex, this only confers a low risk of OSA in the general population. It is unclear why patients with HNC prior to undergoing therapy have such a high rate of OSA, and future research on this topic is warranted. Nevertheless, this study indicates that there is a significantly high risk of OSA among HNC survivors.

OSA is severely detrimental to general health, quality of life, recovery, and potentially increases the risk of cancer.^9^, ^19^, ^32^, ^33^, ^34^, ^35^ Given the findings of this study and risks that OSA poses to HNC survivors, we recommend that post‐treatment HNC patients should undergo routine screening for OSA via well‐validated screening questionnaires. As cancer survivors often struggle with fatigue, this could make the ESS a less useful tool in this specific population. Additionally, recent studies have demonstrated that in general the SBQ is the most accurate OSA screening tool and thus should be the primary screening tool used by providers.^36^ While further research is needed to identify an accurate OSA screener for HNC survivors, we suggest that at this time the SBQ be employed in screening for OSA in HNC patients.

Future research of OSA in HNC survivors should seek to examine the risk of OSA in relation to specific tumor locations, sizes, and stages, as current research is heterogeneous and typically does not compare patients by these categories. Future studies should also control for follow‐up time and assess patients at distinct time intervals. Previous studies have proposed that the development of OSA in HNC survivors could be secondary to radiation‐induced mucosal edema, mucositis, lymphedema, fibrosis, or post‐surgical anatomic alterations.^7^ Continuous follow‐up would therefore allow for evaluation at various stages of healing.

### Limitations

There was significant heterogeneity between included studies regarding tumor location and stage, treatment modality, dose‐dependency of multiple treatment modalities, and the time at which a sleep study was performed after treatment (see Table 3). Many studies did not report AHI for individual patients, which limited data extraction and sub‐group analysis. Additionally, most included studies had small sample sizes. It should also be noted that substantial heterogeneity (>50%) was found which complicates how the results of this study are interpreted.

## Conclusion

This study revealed a high prevalence of OSA in HNC survivors [83.7% (76.0%, 90.3%)] with a statistically significant increase in mean AHI after HNC therapy [4.28 (0.46, 8.09)]. A high prevalence of OSA was noted regardless of HNC treatment modality. Given the risks that OSA poses to HNC survivors regarding wound healing, overall health, quality of life, and potentially cancer recurrence, the authors propose that these patients be routinely evaluated on at least an annual basis for OSA via a well validated instrument such as the STOP‐BANG Questionnaire. Future studies should further investigate risk of OSA in relation to HNC treatment modalities, cancer type/staging, and the accuracy of OSA screening tools in the HNC population.

## Author Contributions

**Om Chitnis**, study design, literature search, data extraction, manuscript writing and editing; **J. Joseph Caraway**, study design, literature search, data extraction, manuscript writing and editing; **Ngun Cer Chin**, study design, literature search, data extraction, manuscript writing and editing; **Anne Guadalupi**, study design, manuscript writing and editing; **Katherine Karahalios**, study design, manuscript writing and editing; **Grace Baisden**, study design, manuscript editing, presentation of research; **Nora Watson**, study design, data analysis, statistical analysis; **Michael Orestes**, study design, literature search, manuscript writing and editing.

## Disclosures

### Competing interests

The contents of this publication are the sole responsibility of the authors and do not necessarily reflect the views, opinions, or policies of The Uniformed Services University of the Health Sciences (USUHS), the Department of Defense (DoD), the Departments of the Army, Navy, or Air Force. Mention of trade names, commercial products, or organizations does not imply endorsement by the US Government.

### Funding source

None.
